# Relationship between exercise induced dyspnea and functional capacity with doppler-derived diastolic function’

**DOI:** 10.1186/1756-0500-6-150

**Published:** 2013-04-15

**Authors:** Sumera Nasim, Najaf Nadeem, Aysha Zahidie, Tabbasum Sharif

**Affiliations:** 1 Department of Cardiology, Orthopaedic and Medical Institute (OMI), Karachi, Pakistan; 2 Section of Cardiology, Department of Medicine, Aga Khan University, Karachi, Pakistan; 3 Department of community Health Sciences, Aga Khan University, Karachi, Pakistan

**Keywords:** Exercise induced dyspnea, Functional capacity, Diastolic dysfunction

## Abstract

**Background:**

Dyspnea is the frequent cause of exercise intolerance and physical inactivity among patients referred for exercise tolerance test. Diastolic dysfunction has shown significant correlation with exercise capacity and exercise induced dyspnea. To find out the frequency of diastolic dysfunction (DD) and the relationships between impaired exercise capacity and exercise induced dyspnea with DD by Doppler-derived indices among patients referred for stress test in a tertiary care hospital of Karachi.

**Methods:**

For this study 135 consecutive patients who were referred for stress test at our non-invasive lab were screened for eligibility. Patients with valvular pathology, atrial fibrillation (AF) and coronary artery disease (CAD) were excluded. Stress test was performed on treadmill using Bruce protocol. Assessment of diastolic function as determined by trans-mitral flow velocity pattern was carried at baseline and at peak exercise. We evaluated impaired exercise capacity and exercise induced dyspnea using validated Borg Scale among study subjects.

**Results:**

Study subjects 88% were males, mean age was 46 ± 16 years, BMI 27 ± 5 kg/m2, prevalence of diabetes mellitus (DM) 15%, hypertension 28% and smoking 21%. Exercise induced DD occurred among 44.6%. Patients with exercise induced DD had lower exercise capacity (9.2 vs. 10.2 METS; p = 0.04) and higher Borg Scale (5.2 vs. 4.0; p < 0.001). DD at baseline was present in 25(26%) of patients so they were excluded from the study. Five patients develop ischemia during stress test so were also excluded. So final analysis was done on 105 patients. Among patients without DD at baseline, there was significant vicariate linear inverse correlation between post exercise E/A ratio and Borg scale (r = −0.23; p = 0.02) and exercise capacity was assessed by exercise duration and MET (r-0.825; p = 0.04). Multivariate regression analysis revealed post exercise E/A ratio as an independent determinant of severity of exercise induced dyspnea and impaired exercise tolerance.

**Conclusion:**

DD is significantly associated with impaired functional capacity and dyspnea among patients referred for exercise tolerance test.

## Background

Cardiac cycle has two phases’ systole phase in which heart contracts and pumps the blood to vital organs of the body and diastolic phase in which heart relaxes. Systolic dysfunction occurs with loss of myocardial tissue and is a known cause of shortness of breath [1]. Diastolic dysfunction includes delayed relaxation, impaired left ventricular (LV) filling and/or increased ventricular stiffness [2]. Diastole has passive filling phase depending on ventricular stiffness and active filling phase that depends on atrial contraction. DD results in a typical upward displacement of the diastolic pressure-volume relationship with increase in end-diastolic pressure, left atrial pressure and pulmo-capillary wedge pressure leading to symptoms of pulmonary congestion [3-5]. Diagnosis of diastolic heart failure requires three conditions (1) presence of signs or symptoms of heart failure; (2) presence of normal or slightly reduced LV ejection fraction (EF > 50%) and (3) presence of increased diastolic filling pressure which can be assessed by echocardiogram [6,7]. Primary diastolic failure is typically seen in patients with hypertensive or valvular heart disease as well as in hypertrophic or restrictive cardiomyopathy but can also occur in a variety of clinical disorders, especially tachycardia and ischemia [8-10]. DD has a particularly high prevalence in elderly patients and is generally associated, with high mortality and morbidity [10,11].

In Framingham study, it was found that patients admitted with heart failure had normal systolic function and were labeled as heart failure patients with normal LV function later on defined as separate entity as Diastolic heart failure [12,13]. Based on this observation, later studies revealed that diastolic dysfunction is also an independent cause of heart failure more frequently in elderly, obese and hypertensive patients [14,15]. The prognosis of diastolic heart failure is usually better than systolic dysfunction [16]. Diastolic heart failure is associated with a lower annual mortality rate of approximately 8% as compared to annual mortality of 19% in heart failure with systolic dysfunction; however, morbidity rate can be substantial [17]. Thus early recognition and appropriate therapy of diastolic dysfunction is worthwhile to prevent progression to diastolic heart failure and subsequent death. Few studies have been conducted to find out the prevalence of diastolic dysfunction in adult Pakistani population presenting with chest pain in health facilities.

Among Pakistani population, dyspnea is the most common end point of patients referred for stress test and physicians don’t know the exact etiology of exercise induce dyspnea among these patients [18]. We built our objectives on the hypothesis that in our patients, diastolic dysfunction is one of the cause of reduced exercise tolerance and shortness of breath during stress test. We used standardized Borg Scale for the assessment of dyspnea as it is a subjective symptom.

### Objectives

1. To find out the frequency of diastolic dysfunction (DD) among patients referred for stress test in a tertiary care hospital of Karachi.

2. To determine the relationship between impaired exercise capacity and dyspnea with DD by Doppler-derived indices.

## Methods

Cross-sectional study design conducted in Outpatient cardiopulmonary department of The Aga Khan University Hospital during Jan 2008-Jan 2009.

### Patient selection

Non probability consecutive sampling technique was used with a sample size of 135 patients meeting the eligibility criteria as below:

#### Inclusion criteria

All adult patients age more than 18 years referred for exercise stress test and had normal diastolic function at baseline. Patients were explained about the study and verbal and written informed consent was taken.

#### Exclusion criteria

1. All patients with known heart failure

2. Patients of CAD,

3. Patients of valvular heart disease

4. Patient with non-sinus rhythm

5. Patient with functional disability

#### Echocardiographic assessment of diastolic function

The ehocardiogaphic assessment of diastolic on trans-thoracic echo used trans-doppler velocities at mitral annulus and pulmonary veins.

### Mitral inflow

The physical principles of conventional pulsed-Doppler focus on the low intensity and high velocity echoes of blood flow. With the imaging plane at the apex 4-chamber view, a sample volume of 2–5 mm is placed at the level of the open leaflets in diastole. Color flow Doppler can be used to help align the sample beam parallel to flow and to locate the point of maximal velocity. Mitral inflow parameters include: early filling peak velocity (E), atrial peak velocity (A), E/A ratio, and deceleration time (DT) or interval between the peak of the E wave to the zero baseline (reflects mean LAP and LV compliance).

### Pulmonary vein flow

The open conduit of flow from the pulmonary veins into the left atrium provides additional information with respect to diastolic function. The left upper pulmonary vein is best suited for Doppler interrogation as it lies nearly parallel to the ultrasound beam. Rotating the probe in the 4-chamber view, the left upper pulmonary vein can be visualized just lateral to the LA appendage. Color flow Doppler is recommended to confirm its location and the presence of laminar flow. The sample volume is placed centrally, 1–2 cm from the vein orifice.

The pulmonary vein systolic flow represents flow into the LA during ventricular systole. In the absence of mitral regurgitation, flow is toward the probe and the spectral display is above the baseline. The pulmonary vein systolic component may appear biphasic. PVS1 represents LA relaxation and the reduction in LA pressure as the base of the heart descends during the early phase of ventricular systole. PVS2, the later systolic peak, reflects right ventricular stroke volume and atrial compliance. The ante grade pulmonary vein diastolic wave (PVD) represents the fall in atrial pressure as the ventricle fills in early diastole, thus, maintaining forward flow from the pulmonary veins into the left atrium.

### Pulsed-doppler patterns of diastolic filling

The four basic transmitral inflow patterns present a parabolic distribution with respect to the E/A ratio. That is as the disease progresses from normal to severe diastolic dysfunction, the E to A relationship changes as follows:

a. Normal (E > A)

b. Abnormal relaxation (E < A)

c. Pseudonormal filling (E > A)

d. Restrictive filling (E>> A)

Trained sonongraphers had performed baseline echocardiogram and systolic and diastolic functions were measured by standard techniques. Eligible patients who were found to have normal baseline diastolic function underwent exercise tolerance test according to full Bruce protocol test. The end point and exercise capacity were noted. Diastolic dysfunction was checked immediately and then also in recovery position of 5–10 minutes. Patients after exercise stress test were discharged home.

#### Exercise stress test

A standard exercise test is performed on treadmill according to standard full bruce protocol with specific indications 1) Asymptomatic patients with valvular heart disease, Pacemaker assessment, Exercise-induced rhythm disturbance evaluation, Patient with Diabetes Mellitus with Age over 35 years or Patient with Cardiac Risks and age >45 years old, Sedentary patient planning new Exercise program.2) Test is also performed among symptomatic patients to assess CAD risk stratification, to assess patient with Exercise-induced dysrhythmia, Patients with Coronary Artery Disease for treatment evaluation, Post-Myocardial Infarction to assess prognosis.

Contraindication include acute myocardial infarction, aortic dissection, critical aortic Stenosis, Critical Left Ventricular outflow-tract obstruct, Inability to exercise to adequate level of exertion unable to perform 5 minutes on Bruce protocol, uninterruptable electrocardiogram, severe uncontrolled hypertension, uncompensated congestive heart failure.

The patient walks on the treadmill, which has a varying speed (which can be altered, i.e. made faster or slower) and a variable gradient (slope), which can mimic going uphill or upstairs. (The Bruce Protocol is a description of the protocol for the increments in speed and gradient in the treadmill test).

During the time of testing, continual monitoring of the patient’s general condition, ECG and blood pressure take place.

A specialist must supervise and full resuscitation facilities must be available. The patient stops when chest pain or discomfort occurs, or when advised to, by the Specialist.

For the Full (Standard) Bruce Protocol, each stage lasts 3 minutes and the speed and gradient are increased at each stage.

The modified test is used in cases where standard testing would be too strenuous for the patient. The patient may not be able to participate in exercise testing because of co-existing problems (i.e. severe OA of the hip, or severe chronic obstructive airways disease).

#### Functional capacity assessment

Functional capacity is the ability of an individual to perform aerobic work as defined by the maximal oxygen uptake (Vo_2max_), that is, the product of cardiac output and arteriovenous oxygen (a − Vo_2_) difference at physical exhaustion, as shown in the following equation:

$${\text{V}}_{02\max}=\left(\text{H}{\text{R}}^{*}\mathrm{SV}\right)\text{a}-{\text{V}}_{02\mathrm{diff}}$$

Usually expressed in metabolic equivalent test (MET).

Where HR indicates heart rate and SV indicates stroke volume.

Although Vo_2max_ is measured in liters of oxygen per minute, it usually is expressed in milliliters of oxygen per kilogram of body weight per minute to facilitate inter-subject comparisons. In addition, functional capacity, particularly when estimated from the work rate achieved rather than directly measured Vo, is frequently expressed in metabolic equivalents (METs), with 1 MET representing the resting energy expenditure (≈3.5 mL O_2_ · kg^−1^ · min^−1^). In this instance, functional capacity is commonly expressed clinically as a multiple of the resting metabolic rate.

Functional capacity, exercise capacity, and exercise tolerance are generally considered synonymous and imply that a maximal exercise test has been performed and maximal effort has been given by the individual. However, these terms also are used occasionally to express an individual’s capacity to perform submaximal activities using one of a variety of tests; therefore, to avoid confusion, the type of exercise evaluation should be specifically described. Functional capacity is usually includes the duration of the exercise period and the workload in METS (metabolic equivalents, or resting oxygen consumption of about 3.5 mL per kg per minute). The interpreter may also add standard described parameters about the patient’s exercise capacity; for example, the report may state “poor exercise tolerance (3 to 4 METS)” or “good exercise tolerance (10 to 11 METS).

### Statistical analysis

Data was double entered on Epi Data version 3.1 and analyzed on SPSS version 19.0. Frequencies were calculated for the dependent and independent variables. Percentage was calculated for categorical variables while for continuous variables means ± standard deviation and ranges were calculated. Prevalence of outcome variables was estimated. The two groups were compared using independent sample t test with 5% level of significance and Confidence interval of 95% was also calculated. The inverse bivariate linear correlation was used to document the correlations of dyspnea and impaired functional capacity with exercise induced diastolic dysfunction. Then multivariate regression analysis was done to adjust for covariates which are clinical parameter like age ,diabetes hypertension BMI and echo derived features like systolic function A and E velocity and ratio of E/A velocities.

### Ethical review

The study was reviewed and approved by the ethical review committee of The Aga Khan University Karachi, Pakistan.

## Results

Data collection was continued for a period of August 2010 till sample size of 105 was achieved. During this period 135 patients presented for exercise stress test. DD at baseline was present in 26% of patients and 5 patients with ischemia on ETT so they were excluded from the study.

The mean age of 105 subjects included in the study was 46 ± 16 yrs, 88% were males and 12% females, mean BMI 27 ± 5 kg/m2. Hypertension was found in 29% patients, hyperlipidemia in 21%, smoking in 27% and diabetes in 21% patients (Table 1).

**Table 1:** The difference in clinical parameters in two groups

**Clinical parameters**	**Total population**	**Exercise induced diastolic dysfunction group (n = 49)**	**Normal diastolic function group (n = 56)**	**P value**
**Mean Age (years)**	46 ± 16	50 ± 12	45 ± 11	0.01
**Mean BMI (kg/m**^**2**^**)**	27 ± 5	28 ± 5	26 ± 6	NS
**Diabetes**	15%	27%	11%	0.05
**Hypertension**	28%	27%	28%	NS
**Dyslipidemia**	10%	8%	13%	NS
**Smoking**	21%	14%	16%	NS

Among study subjects 46% had exercise induced diastolic DD reverted to normal in 10 minutes recovery period. Subjects who had exercise induced DD had significantly lower functional capacity (MET 9.2 vs 10.1 p value 0.04 and mean exercise duration 8 vs 9 p value 0.04) as compared to subjects with normal diastolic function during exercise (Table 2).

**Table 2:** Independent sample t test showing comparison of the exercise induced parameters between two groups of patients

**Parameters**	**Exercise induced DD (n = 49)**	**Normal diastolic function (n = 56)**	**P value**
**Peak Systolic Blood Pressure (mmHg)**	178 ± 8	176 ± 7	NS
**Peak Diastolic Blood Pressure (mm Hg)**	84 ± 4	85 ± 3	NS
**Baseline Heart Rate per minute**	78 ± 8	73 ± 2	NS
**Peak Heart Rate per minute**	167 ± 10	154 ± 5	0.04
**Rate pressure product**	29535 ± 70	27867 ± 58	NS
Mean MET*	9.1 ± 0.6	10.2 ± 1.2	0.04
**Mean Exercise Duration (min)**	8 ± 1	9 ± 2	0.04

*Metabolic equivalency test.

Shortness of the breath or exercise induced dyspnea was one of the common end points of exercise stress test, so we compared shortness of breath using Borg Scale between two groups. Univariate analysis was carried out for independent predictors of shortness of breath as the outcome variable. The independent variable were clinical predictors as age, BMI, gender and echocardiography parameters like E and A wave velocity and E/A ratio were assessed for both the groups. The age (p value 0.01), BMI (p value 0.05) and E/A velocity ratio (p value 0.03) came out to be significantly associated with substantial exercise induced dyspnea (Table 3).

**Table 3:** Univariate analysis of exercise induced dyspnea and functional capacity

**Parameters**	**P value**	**OR(CI)**
**A) Exercise induced Dyspnea**		
Age (years)	0.01	1.3 (0.99-1.5)
DM	0.05	1.2 (0.9-1.4)
Gender	0.09	0.7 (0.4-1.7)
hypertension	0.07	0.6 (0.3-1.8)
BMI (Kg/m^2)^	0.05	1.1(0.7-1.8)
smoking	0.1	0.8 (0.02-1.67)
E velocity	0.03	1.5 (1.1-1.9)
A velocity	0.06	0.9 (0.03-1.9)
E/A velocity	0.001	1.5 (1.1-1.7)
**B) Functional capacity**		
Age(years)	0.06	0.8 (0.5-1.1)
DM	0.05	1.2 (0.9-1.4)
Gender	0.09	0.7 (0.4-1.7)
Hypertension	0.07	0.6 (0.3-1.8)
Smoking	0.1	0.8 (0.02-1.67)
E velocity	0.02	1.5 (1.1-1.9)
A velocity	0.06	0.9 (0.03-1.9)
E/A velocity	0.001	1.5 (1.1-1.7)

We also did univariate analysis of different clinical as well as echocardiography features of DD keeping impaired functional capacity as the outcome. Clinical features as age, gender, comorbids i.e. hypertension, DM, smoking while echocardiography parameters as E velocity, peak A velocity and peak E/A ratio were independent variables were assessed for both the groups. DM and E/A ratio came out to be the strong predictors of impaired functional capacity (p value of 0.05 and 0.02 respectively) due to DD. The E/A ratio of 1.6 was the strongest predictor of impaired functional capacity (p value of <0.01) (Table 3).

On multivariate regression analysis the diastolic dysfunction with diastolic derived parameter E/A velocity ratio was only independent predictor of impaired exercise capacity and exercise induced dyspnea (p value of 0.03) (Table 4).

**Table 4:** Mulvarivate regression analysis of impaired functional capacity

**Parameter**	**P value**	**OR/β(CI)**
**Age**	**0.07**	**0.9 (0.8-1.9)**
**DM**	**0.06**	**0.7 (0.9-1.4)**
**Gender**	**0.09**	**0.7 (0.4-1.7)**
**hypertension**	**0.07**	**0.6 (0.3-1.8)**
**BMI Kg/m2**	**0.07**	**0.7(0.7-1.8)**
**smoking**	**0.1**	**0.8 (0.02-1.67)**
**E velocity**	**0.06**	**0.8 (1.1-1.9)**
**A velocity**	**0.07**	**0.7 (0.03-1.9)**
**E/A velocity**	**0.03**	**1.6 (1.1-1.9)**

### Regression analysis

**Figure 1:**
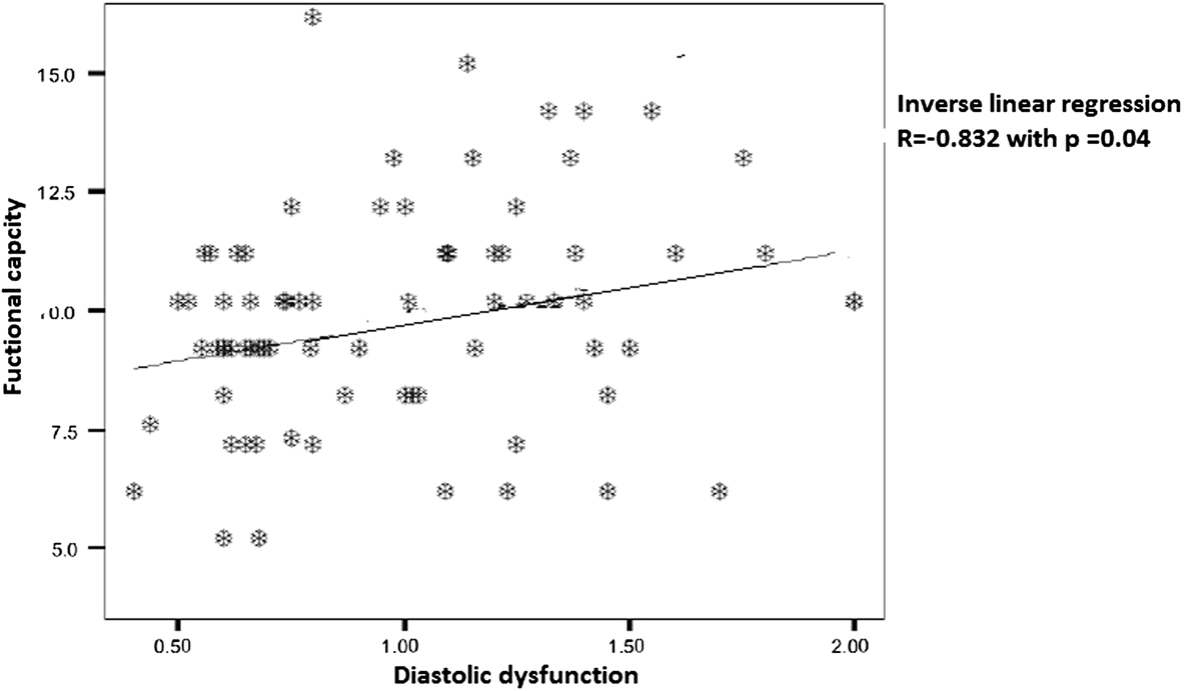
Inverse linear regression of Functional capacity and Diastolic dysfunction.

We found significant inverse bivariate linear correlation of E/A with impaired exercise capacity r of exercise capacity (r-0.825; p = 0.04) (Figure 1).

**Figure 2:**
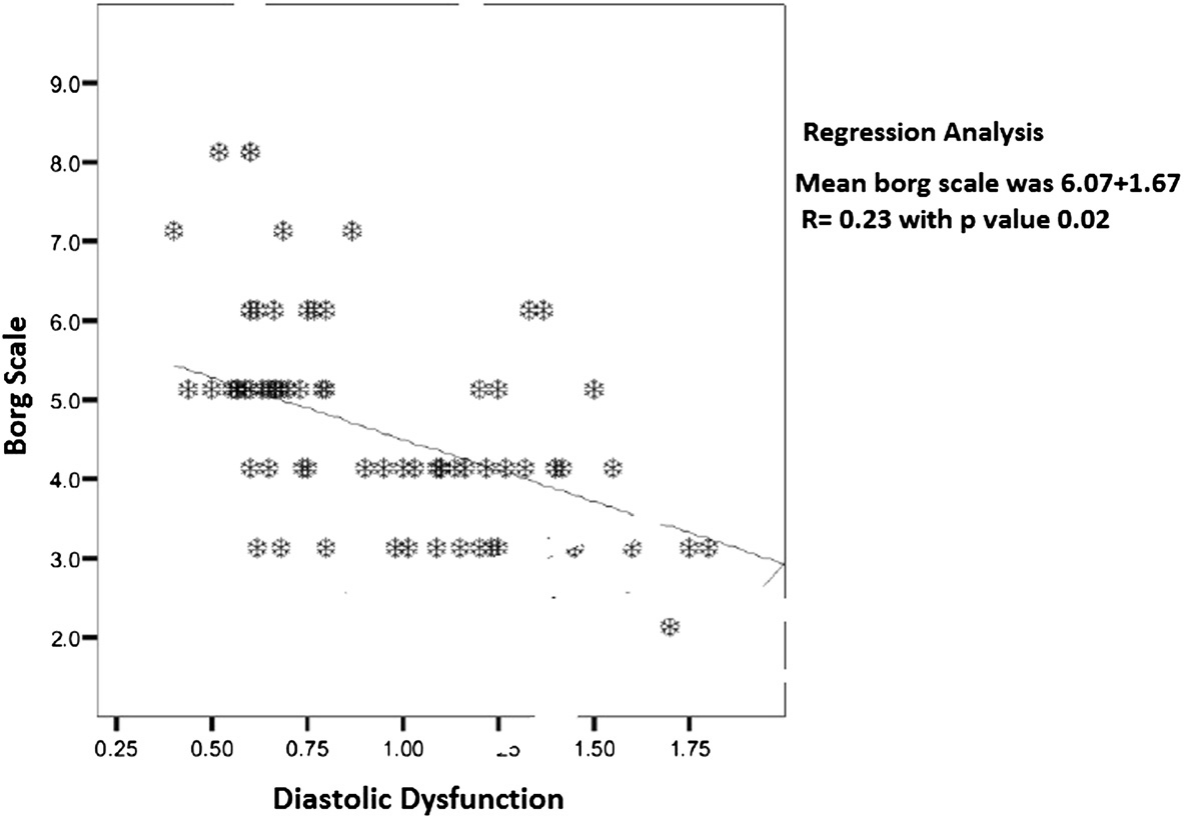
Inverse linear regression of Borg scale with Diastolic dysfunction.

We also found significant inverse bivariate linear correlation of Borg scale with E/A ratio with p value of (r = −0.23; p = 0.02.) (Figure 2).

So on bivariate linear regression analysis diastolic dysfunction is significantly associated with decrease functional capacity and exercise induced dyspnea measured by validated Borg scale.

## Discussion

This study provided the exercise induced diastolic dysfunction among asymptomatic patients in population presenting to a tertiary care hospital in the mega city of Karachi. It was 44% compared to the study by Rashid et al. in which exercise diastolic DD was seen in 48% patients. This difference could be because of the inclusion of high risk population in their study [18]. Our study is the first to describe diastolic dysfunction as cause of exercise induced dyspnea and exercise intolerance among Pakistani population. Among Doppler derived indices, E/A ratio is an independent predictor of exercise induced dyspnea and exercise capacity in our cohort. V.K Mithani et al. carried out study on patients referred for stress echo and found diastolic dysfunction as strong predictor of impaired stress during stress echo [19]. The Studies on patients with severe LV systolic dysfunction revealed DD as an independent predictor of impaired exercise capacity in instead of the low LV Ejection function. DD is also shown to be associated with more ischemia in the myocardial perfusion studies [20-28].

The largest study was by Grewal et al. was conducted on 2867 patients and showed the correlation of diastolic dysfunction as a strong marker of impaired exercise capacity measured in METS [29]. The study explained many factors i.e. advancing age, female sex, greater body-mass index (BMI), and coexisting medical conditions to be associated with decrease in exercise capacity, but elucidating the mechanisms of cardiac-related exercise limitation has been technically difficult to date. Prior research has suggested that measurements of left ventricular systolic function do not predict maximal exercise time in people with normal or impaired left ventricular systolic function [28,29]. Grewal et al. assessed 2867 patients undergoing exercise echocardiography with routine measurements of left ventricular systolic and diastolic dysfunction by two-dimensional and Doppler techniques. DD was strongly and inversely related with exercise capacity. After multivariate adjustment, those with moderate/severe resting DD (−1.30 METs; p < 0.001) and mild resting DD (−0.70 METs; p < 0.001) had substantially lower exercise capacity. Left ventricular filling pressures were similarly associated with a reduction in exercise capacity, each in separate multivariate analyses.

Individuals with impaired relaxation (mild dysfunction) or left ventricular filling pressure E/e’ 15 or greater had a progressive increase in the magnitude of reduction in exercise capacity with advancing age (p < 0.001 and p = 0.02, respectively). Although the results need to be confirmed in prospective trials, even mild abnormalities of diastolic function seemed to be related to a lower exercise capacity and that the magnitude of the effect of diastolic abnormalities became greater in older patients. These points to a potentially modifiable factor that might be a target for interventions that could maintain exercise capacity with aging. Our study also revealed age to be the predictor of shortness of breath in univariate analysis. However in univariate and multivariate analysis age was not a predictor of impaired functional capacity. It could be due to small sample size and diverse population.

Diastolic dysfunction is very common in young population and clinical features usually fail to identify those who will develop DD on exercise. DD is the strong predictor of impaired exercise capacity and shortness of breath. Studies have shown that patients with DD do develop adverse cardiac outcomes on longer follow up [28,29]. We could not correlate this finding clinically because no follow-up was done and it could not be verified whether these patients developed cardiac events and needed treatments to prevent complications for this we need randomized trial.

Our study had some limitation. We used trans-mitral not tissue Doppler velocities for defining DD, transmital velocities are influenced by LV relaxation rate but also by preload, heart rate, age, and LV compliance. Tissue Doppler indices are independent of above mentioned conditions and accurately correlate with LV filling pressure. Other limitations were single Centre experience with small number of patients and cross sectional study design. Due to lack of follow-up we could not assess whether these patients in future would have more clinical cardiac events like heart failure, MI or death.

Clinical utility of outcome of the study includes the fact that even mild abnormalities of diastolic function seemed to be related to a lower exercise capacity along with shortness of breath. Diastolic dysfunction is a potentially modifiable factor that might be a target for interventions that could maintain exercise capacity by early recognition and treatment to avoid long term morbidity and mortality of diastolic dysfunction. However we need a long term follow up study to strengthen the evidence of this observational study.

## Conclusion

The diastolic dysfunction alone during exercise is the strong predictor of impaired exercise capacity and shortness of breath. Diastolic dysfunction is very common in our younger asymptomatic population and we need large prospective studies to determine whether members of an apparently healthy population with diastolic dysfunction will remain asymptomatic or eventually develop heart failure or other cardiac events.

## Competing interests

The authors declare that they have no competing interests.

## Authors’ contributions

SN conceived the study and supervised the data collection. NN and AZ performed the statistical analysis. SN, NN, AZ and TS drafted and revised the manuscript. All authors read and approved the final draft.
